# Supplementary material for: External validation of EPIC’s Risk of Unplanned Readmission model, the LACE+ index and SQLape as predictors of unplanned hospital readmissions: A monocentric, retrospective, diagnostic cohort study in Switzerland
Source: PLoS One. 2021 Nov 12;16(11):e0258338. doi: 10.1371/journal.pone.0258338 (PMC8589185; doi:10.1371/journal.pone.0258338)
Supplement: S1 Appendix — (DOCX) [file pone.0258338.s001.docx]

# **S1. Appendix**

## **Anatomical Therapeutic Chemical Classification System (ATC) - Subgroups**

| **Medication groups (English)** | **ATC Code** | **Grouper** |
| --- | --- | --- |
| Vitamin K-Antagonist | B01AA | Anticoagulants |
| Heparin-Group | B01AB | Anticoagulants |
| Platelet aggregation inhibitor, excl. Heparin | B01AC | Anticoagulants |
| Enzyme (B01AD) | B01AD | Anticoagulants |
| Direct Thrombin Inhibitors | B01AE | Anticoagulants |
| Direct Factor Xa-Inhibitors | B01AF | Anticoagulants |
| Other antithrombotic drugs | B01AX | Anticoagulants |
| Blood transfusions, excipients | V07AC | Anticoagulants |
| Butylpyrazolidines | M01AA | Non-steroidal anti-inflammatories |
| Acetic acid derivatives and related substances | M01AB | Non-steroidal anti-inflammatories |
| Oxicame | M01AC | Non-steroidal anti-inflammatories |
| Propionic acid derivatives | M01AE | Non-steroidal anti-inflammatories |
| Fenamate | M01AG | Non-steroidal anti-inflammatories |
| Coxibe selective cyclooxygenase-2 inhibitors | M01AH | Non-steroidal anti-inflammatories |
| Other non-steroidal anti-inflammatory and anti-rheumatic drugs | M01AX | Non-steroidal anti-inflammatories |
| Mineralocorticoids | H02AA | Corticosteroids |
| Glucocorticoids | H02AB | Corticosteroids |
| Corticosteroids for systemic use, combinations | H02BX | Corticosteroids |
| Corticosteroids, weakly active (Group I) | D07AA | Corticosteroids |
| Corticosteroids, moderately active (Group II) | D07AB | Corticosteroids |
| Corticosteroids, potent (Group III) | D07AC | Corticosteroids |
| Corticosteroids, very potent (Group IV) | D07AD | Corticosteroids |
| Corticosteroids, weakly effective, combinations with antiseptics | D07BA | Corticosteroids |
| Corticosteroids, medium strength, combinations with antiseptics | D07BB | Corticosteroids |
| Corticosteroids, highly effective, combinations with antiseptics | D07BC | Corticosteroids |
| Corticosteroids, very potent, combinations with antiseptics | D07BD | Corticosteroids |
| Corticosteroids, weakly effective, combinations with antibiotics | D07CA | Corticosteroids |
| Corticosteroids, moderately effective, combinations with antibiotics | D07CB | Corticosteroids |
| Corticosteroids, highly effective, combinations with antibiotics | D07CC | Corticosteroids |
| Corticosteroids, very potent, combinations with antibiotics | D07CD | Corticosteroids |
| Corticosteroids, weakly effective, other combinations | D07XA | Corticosteroids |
| Corticosteroids, medium strength, other combinations | D07XB | Corticosteroids |
| Corticosteroids, potent, other combinations | D07XC | Corticosteroids |
| Corticosteroids, very potent, other combinations | D07XD | Corticosteroids |
| Antibiotics and corticosteroids | G01BA | Corticosteroids |
| Quinoline derivatives and corticosteroids | G01BC | Corticosteroids |
| Antiseptics and corticosteroids | G01BD | Corticosteroids |
| Sulfonamides and corticosteroids | G01BE | Corticosteroids |
| Imidazole derivatives and corticosteroids | G01BF | Corticosteroids |
| Corticosteroids (S02BA) | S02BA | Corticosteroids |
| Corticosteroids and anti-infectives in combination (S02CA) | S02CA | Corticosteroids |
| Corticosteroids (S03BA) | S03BA | Corticosteroids |
| Corticosteroids and anti-infectives in combination (S03CA) | S03CA | Corticosteroids |
| Phenothiazines with aliphatic side chains | N05AA | Antipsychotics |
| Phenothiazines with piperazine structure | N05AB | Antipsychotics |
| Phenothiazines with piperidine structure | N05AC | Antipsychotics |
| Butyrophenone derivatives | N05AD | Antipsychotics |
| Indole derivatives | N05AE | Antipsychotics |
| Thioxanthene derivatives | N05AF | Antipsychotics |
| Diphenylbutylpiperidine derivatives | N05AG | Antipsychotics |
| Diazepin, Oxazepin, Thiazepin und Oxepin | N05AH | Antipsychotics |
| Neuroleptics in late dyskinesia | N05AK | Antipsychotics |
| Benzamides | N05AL | Antipsychotics |
| Lithium | N05AN | Antipsychotics |
| Other antipsychotics | N05AX | Antipsychotics |
| Antidepressants in combination with psycholeptics | N06CA | Antipsychotics |
| Fatty acid derivatives | N03AG | Antipsychotics |
| Carboxamid-Derivat | N03AF | Antipsychotics |
| Histamine H2-receptor antagonists | A02BA | Ulcer medications |
| Prostaglandins | A02BB | Ulcer medications |
| Proton pump inhibitors | A02BC | Ulcer medications |
| Combinations for the eradication of Helicobacter pylori | A02BD | Ulcer medications |
| Other agents for peptic ulcer and gastrooesophageal reflux disease | A02BX | Ulcer medications |
