# Supplementary material for: External validation of EPIC’s Risk of Unplanned Readmission model, the LACE+ index and SQLape as predictors of unplanned hospital readmissions: A monocentric, retrospective, diagnostic cohort study in Switzerland
Source: PLoS One. 2021 Nov 12;16(11):e0258338. doi: 10.1371/journal.pone.0258338 (PMC8589185; doi:10.1371/journal.pone.0258338)
Supplement: S2 Appendix — (DOCX) [file pone.0258338.s002.docx]

# **S2. Appendix**

## **Laboratory components and corresponding reference ranges**

| **Record Name** | **Record Name (German)** |  | **Indicator** | **Unit** | **Reference range** | **Dorner Laboratory System Identifier** |
| --- | --- | --- | --- | --- | --- | --- |
| BUN | Harnstoff |  | HIGH | mg/dL | 106.729 – 313.227 | CUREA |
| Calcium | Calcium |  | LOW | mg/dL | 85.074 – 98.609 | CCA |
| Creatinine | Creatinin (enzymatisch) |  | HIGH | mg/dL | 0.6667 – 1.1752 | CCREA |
| Creatinine | Creatinin (Elektrode) |  | HIGH | mg/dL | 0.5989 – 1.1978 | CREA-ABL |
| Hemoglobin | Hämoglobin |  | LOW | g/dL | 115 - 148 | HGHB |
| PT/INR | INR korrigiert |  | HIGH | Ratio | < 1.3 | HSINRHK |
| PT/INR | INR |  | HIGH | Ratio | < 1.3 | HS1INR |
| PT/INR | INR |  | HIGH | Ratio | 1 – 1.3 | HSINR |
| Phosphorus | Phosphat |  | Tested Yes | - | - | CPHOS |

**Abbreviations:** BUN = blood urea nitrogen; PT/INR = prothrombin time and international normalized ratio; mg/dL = milligrams per deciliter; g/dL = grams per deciliter; denoted reference ranges are exemplary and may have varied dependent on reagent and laboratory test
