# Supplementary material for: External validation of EPIC’s Risk of Unplanned Readmission model, the LACE+ index and SQLape as predictors of unplanned hospital readmissions: A monocentric, retrospective, diagnostic cohort study in Switzerland
Source: PLoS One. 2021 Nov 12;16(11):e0258338. doi: 10.1371/journal.pone.0258338 (PMC8589185; doi:10.1371/journal.pone.0258338)
Supplement: S3 Appendix — (DOCX) [file pone.0258338.s003.docx]

# **S3. Appendix**

## **Detailed description of prediction model variables**

**EPIC´s Risk of Unplanned Readmission model**

| **Data category & type** | **Variable name** | **Given definition / stated in the development study/by the developers** | **Working definition (Cohort A)** | **Working definition (Cohort B)** | **Point in time of data collection** |
| --- | --- | --- | --- | --- | --- |
| Demographics, numeric | Age | Age at the day of hospital admission | Age at the day of hospital admission | same as given definition | Retrospectively |
| Administrative data, numeric | Current length of stay | The number of days of hospitalization of the ongoing stay, from the time of inpatient admission till the point in time of score calculation. It does not consider any time spent in the Emergency department/any time spent prior to inpatient admission. Rounded to 3 decimal points. (Time stamp at model calculation - admission time stamp). | The number of days of hospitalization of the ongoing stay, from the time of inpatient admission till the point in time of score calculation. It **does** consider the time spent in the Emergency department. Rounded to 3 decimal points. (Time stamp at model calculation - hospital admission time stamp). | same as given definition | Retrospectively |
| Resource utilization, numeric | Number of past ED visits, in the last 6 months | A count of the number of ED visits in the last six months. Counts both, those where the patient went home healthy, and the ones where they were subsequently submitted to the wards. Lookback starts at the day of admission | same as given definition | same as given definition | Retrospectively |
| Medications, numeric | Number of active medication orders | Number of active medications of a patient at the point in time of score calculation. The number of active medications includes the entry/outpatient and the inpatient medications. Discontinued medications are excluded. Counts prescribed medication on demand.  Example: If a patient arrives while taking a beta-blocker (discontinued during the stay), and is sent home with the same beta-blocker, that would be only one order. If a patient is also given THE SAME beta-blocker as an Inpatient order, they would have a count of 2 for that medication during the time while they are taking the medication on an inpatient unit.   Prescriptions of the same medication but as varying dosage on the same day count as one prescription/active medication.  Several prescriptions of the same medication on the same day no matter the dosage count as one prescription/active medication.  Prescriptions of the same active ingredient but through various routes of administration (orally, intravenously, etc.) count as separate prescriptions.  Prescriptions of the same active ingredient but as different medicinal products count as separate prescriptions. | **Total number** of prescribed medications **during** the hospital stay at discharge.   Does include patient's medication on demand only if administered. Does **not** include entry/outpatient medication.   Several prescriptions of the same medication **with the same dosage** count as one prescription/active medication.  Prescriptions of the same medication but as varying dosage on the same day count **separately**.  Prescriptions of the same active ingredient but through various routes of administration (orally, intravenously, etc.) count as separate prescriptions.  Prescriptions of the same active ingredient but as different medicinal products count as separate prescriptions. | same as given definition | Retrospectively |
| Resource utilization, numeric | Number of past admissions in the last 12 months | Count of the number of inpatient stays in the last 12 months. It includes hospitalizations no matter how many days the patient has stayed (the patient does not need to stay for the night; admission and same day discharge stays are included). ED visits without transfer to the ward and ambulant office visits are excluded. The admission type (urgent, elective, etc.) is not relevant. Lookback period starts at the day of admission. | same as given definition | same as given definition | Retrospectively |
| Resource utilization, categorical | Has future scheduled appointments | Does the patient have an outpatient appointment scheduled for any time after today? Planned hospital stays are not counted. There is no maximum look forward period. Any scheduled appointment in the future will be considered. | same as given definition | same as given definition | Retrospectively |
| **Data category & type** | **Variable name** | **Given definition / stated in the development study/by the developers** | **Working definition (Cohort A)** | **Working definition (Cohort B)** | **Point in time of data collection** |
| Resource utilization, categorical | Prior length of stay of 10 days or more in the last 12 months | Has the patient had a hospital stay of at least 10 days in the last 12 months. Lookback period starts at the day of admission. | same as given definition | same as given definition | Retrospectively |
| Comorbidities, categorical | Diagnosis of cancer | At the time of score calculation, has the patient had a diagnosis from ICD-10 CM grouper Diagnosis of Cancer? | At discharge, has the patient had a diagnosis listed in the corresponding ICD-10 GM grouper? The original groupers were replicated containing mapped ICD-10 codes from the German modification (GM). The exact codes are available from the corresponding author upon reasonable request. | same as given definition, but mapped onto ICD-10 GM | Retrospectively |
| Comorbidities, categorical | Diagnosis of Deficiency Anemia | At the time of score calculation, has the patient had a diagnosis from ICD-10 CM grouper Deficiency Anemia? | At discharge, has the patient had a diagnosis listed in the corresponding ICD-10 GM grouper? The original groupers were replicated containing mapped ICD-10 codes from the German modification (GM). The exact codes are available from the corresponding author upon reasonable request. | same as given definition, but mapped onto ICD-10 GM | Retrospectively |
| Comorbidities, categorical | Diagnosis of Electrolyte Disorder | At the time of score calculation, has the patient had a diagnosis from ICD-10 CM grouper Electrolyte Disorder? | At discharge, has the patient had a diagnosis listed in the corresponding ICD-10 GM grouper? The original groupers were replicated containing mapped ICD-10 codes from the German modification (GM). The exact codes are available from the corresponding author upon reasonable request. | same as given definition, but mapped onto ICD-10 GM | Retrospectively |
| Comorbidities, categorical | Diagnosis of Renal Failure | At the time of score calculation, has the patient had a diagnosis from ICD-10 CM grouper Diagnosis of Renal Failure? | At discharge, has the patient had a diagnosis listed in the corresponding ICD-10 GM grouper? The original groupers were replicated containing mapped ICD-10 codes from the German modification (GM). The exact codes are available from the corresponding author upon reasonable request. | same as given definition, but mapped onto ICD-10 GM | Retrospectively |
| Comorbidities, categorical | Diagnosis of Drug Abuse | At the time of score calculation, has the patient had a diagnosis from ICD-10 CM grouper Diagnosis of Drug Abuse? | At discharge, has the patient had a diagnosis listed in the corresponding ICD-10 GM grouper? The original groupers were replicated containing mapped ICD-10 codes from the German modification (GM). The exact codes are available from the corresponding author upon reasonable request. | same as given definition, but mapped onto ICD-10 GM | Retrospectively |
| Biological data, categorical | Hemoglobin | For lab values, at the time of score calculation, most recent lab results from the last 72 hours are being compared to the corresponding reference range to determine if it is high/low/normal. | For lab values, at the time of score calculation, most recent lab results from the last 72 hours are being compared to the corresponding reference range to determine if it is high/low/normal.  For biological data, variables hemoglobin, calcium, BUN, etc. were matched with the local system's laboratory components. The process was done by an experienced Lab. Analyst. Please see "Lab. Components" sheet for the exact components used to identify relevant lab values. | same as working definition in column G | Retrospectively |
| Biological data, categorical | Calcium | For lab values, at the time of score calculation, most recent lab results from the last 72 hours are being compared to the corresponding reference range to determine if it is high/low/normal. | For lab values, at the time of score calculation, most recent lab results from the last 72 hours are being compared to the corresponding reference range to determine if it is high/low/normal.  For biological data, variables hemoglobin, calcium, BUN, etc. were matched with the local system's laboratory components. The process was done by an experienced Lab. Analyst. Please see "Lab. Components" sheet for the exact components used to identify relevant lab values. | same as working definition in column G | Retrospectively |
| **Data category & type** | **Variable name** | **Given definition / stated in the development study/by the developers** | **Working definition (Cohort A)** | **Working definition (Cohort B)** | **Point in time of data collection** |
| Biological data, categorical | Blood Urea Nitrogen (BUN) | For lab values, at the time of score calculation, most recent lab results from the last 72 hours are being compared to the corresponding reference range to determine if it is high/low/normal. | For lab values, at the time of score calculation, most recent lab results from the last 72 hours are being compared to the corresponding reference range to determine if it is high/low/normal.  For biological data, variables hemoglobin, calcium, BUN, etc. were matched with the local system's laboratory components. The process was done by an experienced Lab. Analyst. Please see "Lab. Components" sheet for the exact components used to identify relevant lab values. | same as working definition in column G | Retrospectively |
| Biological data, categorical | Creatinine | For lab values, at the time of score calculation, most recent lab results from the last 72 hours are being compared to the corresponding reference range to determine if it is high/low/normal. | For lab values, at the time of score calculation, most recent lab results from the last 72 hours are being compared to the corresponding reference range to determine if it is high/low/normal.  For biological data, variables hemoglobin, calcium, BUN, etc. were matched with the local system's laboratory components. The process was done by an experienced Lab. Analyst. Please see "Lab. Components" sheet for the exact components used to identify relevant lab values. | same as working definition in column G | Retrospectively |
| Biological data, categorical | Phosphate | Has the patient had a phosphate lab in the last 3 days? Lookback period starts at the point in time of score calculation. | For lab values, at the time of score calculation, most recent lab results from the last 72 hours are being compared to the corresponding reference range to determine if it is high/low/normal.  For biological data, variables hemoglobin, calcium, BUN, etc. were matched with the local system's laboratory components. The process was done by an experienced Lab. Analyst. Please see "Lab. Components" sheet for the exact components used to identify relevant lab values. | same as working definition in column G | Retrospectively |
| Biological data, categorical | Prothrombin Time and International Normalized Ratio (PT/INR) | For lab values, at the time of score calculation, most recent lab results from the last 72 hours are being compared to the corresponding reference range to determine if it is high/low/normal. | For lab values, at the time of score calculation, most recent lab results from the last 72 hours are being compared to the corresponding reference range to determine if it is high/low/normal.  For biological data, variables hemoglobin, calcium, BUN, etc. were matched with the local system's laboratory components. The process was done by an experienced Lab. Analyst. Please see "Lab. Components" sheet for the exact components used to identify relevant lab values. | same as working definition in column G | Retrospectively |
| Medications, | Anticoagulants | At the time of score calculation, has the patient had active orders from certain ATC groups? | At the time of score calculation, has the patient had active orders from certain ATC groups? Based on the original specifications and with regard to local regulations and classifications, an experienced pharmacist developed Swiss specific ATC groups. Please see "ATC-Codes" sheet for the exact codes used to identify relevant medications. | same as working definition in column G | Retrospectively |
| Medications, categorical | Non-Steroidal Anti-Inflammatory drugs | At the time of score calculation, has the patient had active orders from certain ATC groups? | At the time of score calculation, has the patient had active orders from certain ATC groups? Based on the original specifications and with regard to local regulations and classifications, an experienced pharmacist developed Swiss specific ATC groups. Please see "ATC-Codes" sheet for the exact codes used to identify relevant medications. | same as working definition in column G | Retrospectively |
| Medications, categorical | Corticosteroids | At the time of score calculation, has the patient had active orders from certain ATC groups? | At the time of score calculation, has the patient had active orders from certain ATC groups? Based on the original specifications and with regard to local regulations and classifications, an experienced pharmacist developed Swiss specific ATC groups. Please see "ATC-Codes" sheet for the exact codes used to identify relevant medications. | same as working definition in column G | Retrospectively |
| Medications, categorical | Antipsychotics | At the time of score calculation, has the patient had active orders from certain ATC groups? | At the time of score calculation, has the patient had active orders from certain ATC groups? Based on the original specifications and with regard to local regulations and classifications, an experienced pharmacist developed Swiss specific ATC groups. Please see "ATC-Codes" sheet for the exact codes used to identify relevant medications. | same as working definition in column G | Retrospectively |
| **Data category & type** | **Variable name** | **Given definition / stated in the development study/by the developers** | **Working definition (Cohort A)** | **Working definition (Cohort B)** | **Point in time of data collection** |
| Medications, categorical | Ulcer Medication | At the time of score calculation, has the patient had active orders from certain ATC groups? | At the time of score calculation, has the patient had active orders from certain ATC groups? Based on the original specifications and with regard to local regulations and classifications, an experienced pharmacist developed Swiss specific ATC groups. Please see "ATC-Codes" sheet for the exact codes used to identify relevant medications. | same as working definition in column G | Retrospectively |
| Interventions/Orders, categorical | Imaging Orders | Has the patient had an order of this type in the last six months? Lookback period starts at the day of admission. | Has the hospital provided an order of this type to the patient in the last six months? / Has the hospital documented any related "tarif medical" (TARMED) service codes of the TARMED chapter 39 (catalogue version 1.09, valid from 01.01.2018)? | same as working definition in column G | Retrospectively |
| Interventions/Orders, categorical | Electrocardiography (ECG) | Has the patient had an order of this type in the last six months? Lookback period starts at the day of admission. | Has the hospital provided an order of this type to the patient in the last six months? / Has the hospital documented any related "tarif medical" (TARMED) service codes of the following (catalogue version 1.09, valid from 01.01.2018): -17.0010 Electrocardiogram(ECG) -17.0080 Exercise ECG -17.0090 Exercise ECG, Ergometry -17.0120 ECG rhythm strip, per 5 minutes -17.0130 ECG, Attach incl. remove | same as working definition in column G | Retrospectively |
| Interventions/Orders, categorical | Restraining Orders | Has the patient had an order of this type in the last six months? Lookback period starts at the day of admission. | Not relevant, has not occurred in 2018 at the site. | Not relevant, has not occurred in 2018 at the site. | Retrospectively |
| Comorbidities, numeric | Charlson Comorbidity Score (EPIC version) | To calculate the adapted Charlson Comorbidity Score the following formula was used: Charlson Comorbidity Score- Between 0 and 32, based on these diagnoses: 1 pt. - Myocardial Infarction 1 pt. - Peripheral Vascular Disease 1 pt. - Cerebrovascular Disease 1 pt. - Diabetes w/o chronic complications 2 pt. - Cancer 2 pt. - Mild Liver Disease 2 pt. - Chronic Pulmonary Disease 2 pt. - Congestive Heart Failure 3 pt. - Dementia 3 pt. - Rheumatic Disease 4 pt. – HIV/AIDS 4 pt. - Moderate or Severe Liver Disease 6 pt. - Metastic Solid Tumor The Charlson Score was calculated on the time of the readmission score calculation. | The original groupers were replicated containing mapped ICD-10 codes from the German modification (GM). Please see "ICD_10_GM_Codes" sheet for the exact codes used to identify relevant disorders. The comorbidity score was calculated based on all known diagnoses at the day of discharge. For this study the working assumption was established, that after establishing admission diagnosis, diagnoses do not change throughout the hospital stay (admission = discharge diagnoses). | same as given definition but mapped onto ICD-10 GM | Retrospectively |

***© 2020 Epic Systems Corporation***

**SQLape®**

| **Data category & type** | **Variable name** | **Given definition / stated in the development study/by the developers** | **Working definition (Cohort A)** | **Working definition (Cohort B)** | **Point in time of data collection** |
| --- | --- | --- | --- | --- | --- |
| Comorbidities | SQLape diagnosis groups | The expected rates of potentially avoidable readmissions were estimated using a licensed plugin. The expected rates are being calculated based on the Medical Statistics of Hospitals dataset. Variable specifications can be found online: https://www.bfs.admin.ch/bfs/de/home/statistiken/gesundheit/erhebungen/ms.html. For more information regarding the SQLape algorithm, please check http://www.sqlape.com/readmissions/. | same as given definition | not relevant | Retrospectively |
| Interventions/Orders | SQLape surgical intervention groups |  | same as given definition | not relevant | Retrospectively |
| Demographics | Age |  | same as given definition | not relevant | Retrospectively |
| Resource utilization | Previous hospitalization during the six months before the index discharge |  | same as given definition | not relevant | Retrospectively |
| Resource utilization | Planned hospitalization |  | same as given definition | not relevant | Retrospectively |
| Comorbidities | Complexity |  | same as given definition | not relevant | Retrospectively |

**LACE+**

| **Data category & type** | **Variable name** | **Given definition / stated in the development study/by the developers** | **Working definition (Cohort A)** | **Working definition (Cohort B)** | **Point in time of data collection** |
| --- | --- | --- | --- | --- | --- |
| Demographics | Gender (male) | Gender | Gender | not relevant | Retrospectively |
| Administrative data | Urgent admission | Urgent admission | Emergency (treatment within 12 hours indispensable) | not relevant | Retrospectively |
| Administrative data | Discharge institution (Teaching vs small non-teaching hospital) | Small nonteaching hospital = nonteaching hospital with < 100 beds, large nonteaching hospital = nonteaching hospital ≥ 100 beds | Small nonteaching hospital = nonteaching hospital with < 100 beds, large nonteaching hospital = nonteaching hospital ≥ 100 beds | not relevant | Retrospectively |
| Administrative data | Discharge institution (Large vs small non-teaching hospital) | Small nonteaching hospital = nonteaching hospital with < 100 beds, large nonteaching hospital = nonteaching hospital ≥ 100 beds | Small nonteaching hospital = nonteaching hospital with < 100 beds, large nonteaching hospital = nonteaching hospital ≥ 100 beds | not relevant | Retrospectively |
| Demographics | Age | Age at the day of hospital admission | Age at the day of hospital admission | not relevant | Retrospectively |
| Administrative data | Length of stay | Current length of stay, at the day of model calculation | The number of days of hospitalization of the ongoing stay, from the time of inpaitent admission. Therefore, it does consider any time spent in the Emergency department/any time spent prior to inpatient admission. Rounded to 3 decimal points. (Time stamp at model calculation - admission time stamp). | not relevant | Retrospectively |
| Comorbidities | Charlson Comorbidity Score | The Charlson score was calculated from diagnoses for the index admission and the International Statistical Classification of Diseases and Related Health Problems, 10th revision, Canada (ICD-10-CA) codes cited by Quan and colleagues.  Quan H, Sundararajan V, Halfon P, Fong A, Burnand B, Luthi JC, et al. Coding algorithms for defining comorbidities in ICD-9-CM and ICD-10 administrative data. Med Care 2005;43(11):1130–1139 | The Charlson score was calculated from diagnoses for the index admission and the International Statistical Classification of Diseases and Related Health Problems, 10th revision, Canada (ICD-10-CM) codes cited by Quan and colleagues but adapted for the use in Switzerland (ICD-10-CM codes were mapped to ICD-10-GM codes). For more details, please see the excel sheet "ICD_10_GM_Codes".  Quan H, Sundararajan V, Halfon P, Fong A, Burnand B, Luthi JC, et al. Coding algorithms for defining comorbidities in ICD-9-CM and ICD-10 administrative data. Med Care 2005;43(11):1130–1139 | not relevant | Retrospectively |
| Resource utilization | Number of ED visits in the previous 6 months | A count of the number of ED visits in the last six months. Counts both those where the patient went home healthy, and the ones where they were subsequently submitted to the wards. Lookback starts at the day of admission | A count of the number of ED visits in the last six months. Counts both those where the patient went home healthy, and the ones where they were subsequently submitted to the wards. Lookback starts at the day of admission | not relevant | Retrospectively |
| Resource utilization | Number of urgent admissions in previous year | A count of the number of urgent hospital admissions, Lookback starts at the day of admission | A count of the number of urgent hospital admissions (through ED) Lookback starts at the day of admission | not relevant | Retrospectively |
| Resource utilization | Number of elective admissions in previous year | A count of the number of elective hospital admissions, Lookback starts at the day of admission | A count of the number of elective hospital admissions, Lookback starts at the day of admission | not relevant | Retrospectively |
| Comorbidities | CMG score | Case Mix Group (CMG) variable is only available in Canada | Case Mix Group (CMG) variable is only available in Canada | not relevant | Retrospectively |
| **Data category & type** | **Variable name** | **Given definition / stated in the development study/by the developers** | **Working definition (Cohort A)** | **Working definition (Cohort B)** | **Point in time of data collection** |
| Administrative data | Number of days on ALC status | Number of days on alternative level of care (ALC) status | Alternative level of care status stands for patients who stay at the hospital but no longer receive active medical care, coded as main diagnosis Z75.8 ICD-10 code | not relevant | Retrospectively |
| Other | Interaction term 1 (Age x Charlson score) | please see age and Charlson score | please see age and Charlson score | not relevant | Retrospectively |
| Other | Interaction term 2 (Age x Number of urgent admissions in previous year) | please see age and number of urgent admissions in previous year | please see age and number of urgent admissions in previous year | not relevant | Retrospectively |
| Other | Interaction term 3 (Charlson score x Number of urgent admissions in previous year) | please see Charlson score and number of urgent admissions in previous year | please see Charlson score and number of urgent admissions in previous year | not relevant | Retrospectively |

| **Color coding** |  |
| --- | --- |
| Same working definition as specified in the original study | |
| Adaptations to the Swiss setting | |
| Slight modifications |  |
| Extensive modifications |  |
