# Supplementary material for: External validation of EPIC’s Risk of Unplanned Readmission model, the LACE+ index and SQLape as predictors of unplanned hospital readmissions: A monocentric, retrospective, diagnostic cohort study in Switzerland
Source: PLoS One. 2021 Nov 12;16(11):e0258338. doi: 10.1371/journal.pone.0258338 (PMC8589185; doi:10.1371/journal.pone.0258338)
Supplement: S4 Appendix — (DOCX) [file pone.0258338.s004.docx]

# **S4. Appendix**

## **Cohort B – Brier scores**

|  | Cohort B  Sept. 23, 2019 – Dec. 31, 2019 | | | |
| --- | --- | --- | --- | --- |
| Scores  (day, time) | **Patients/scores N** | **with readmission N** (%) | **Brier score** |  |
|  |  |  |  |  |
| EPIC’s score,  admission day (8 a.m.) | 1233 | 32  (2.6) | 0.02598 |  |
| EPIC’s score,  admission day (12 a.m.) | 3217 | 97  (3.0) | 0.02944 |  |
| EPIC’s score,  1^st^ day (8 a.m.) | 6787 | 277  (4.1) | 0.03925 |  |
| EPIC’s score,  1^st^ day (12 a.m.) | 6567 | 278  (4.2) | 0.04064 |  |
| EPIC’s score,  2^nd^ day (8.a.m.) | 5935 | 259  (4.4) | 0.04185 |  |
| EPIC’s score,  2^nd^ day (12 a.m.) | 5233 | 233  (4.4) | 0.04273 |  |
| EPIC’s score,  3^rd^ day (8 a.m.) | 4273 | 199  (4.7) | 0.04471 |  |
| EPIC’s score,  3^rd^ day (12 a.m.) | 3707 | 184  (5.0) | 0.04742 |  |
| EPIC’s score,  4^th^ day (8 a.m.) | 2976 | 162  (5.4) | 0.05140 |  |
| EPIC’s score,  4^th^ day (12 a.m.) | 2593 | 152  (5.9) | 0.05514 |  |
| EPIC’s score,  5^th^ day (8 a.m.) | 2100 | 134  (6.4) | 0.04973 |  |
| EPIC’s score,  5^th^ day (12 a.m.) | 1875 | 124  (6.6) | 0.06186 |  |
| EPIC’s score,  day before discharge (8 a.m.) | 6259 | 273  (4.4) | 0.04208 |  |
| EPIC’s score,  day before discharge (12 a.m.) | 6530 | 280  (4.3) | 0.04149 |  |
| EPIC’s score,  discharge day (8 a.m.) | 7071 | 303  (4.3) | 0.04150 |  |
| EPIC’s score,  discharge day (12 a.m.) | 4234 | 187  (4.4) | 0.04262 |  |
