# Supplementary material for: External validation of EPIC’s Risk of Unplanned Readmission model, the LACE+ index and SQLape as predictors of unplanned hospital readmissions: A monocentric, retrospective, diagnostic cohort study in Switzerland
Source: PLoS One. 2021 Nov 12;16(11):e0258338. doi: 10.1371/journal.pone.0258338 (PMC8589185; doi:10.1371/journal.pone.0258338)

# **S5. Appendix**

## **Calibration plots by risk group thresholds, Cohort A**

| No risk: | 0 – baseline* | [0 – 5.1%] |
| --- | --- | --- |
| Low risk: | Baseline - 2 x baseline | (5.1% - 10.2%] |
| Medium risk: | 2 x baseline – 3 x baseline | (10.2% – 15.3%] |
| High risk: | >= 3 x baseline | (15.3% - 100%] |

*baseline risk corresponds to the prevalence of unplanned readmissions within 30 days in Cohort A (5.1%)

**EPIC’s Risk of Unplanned Readmission**


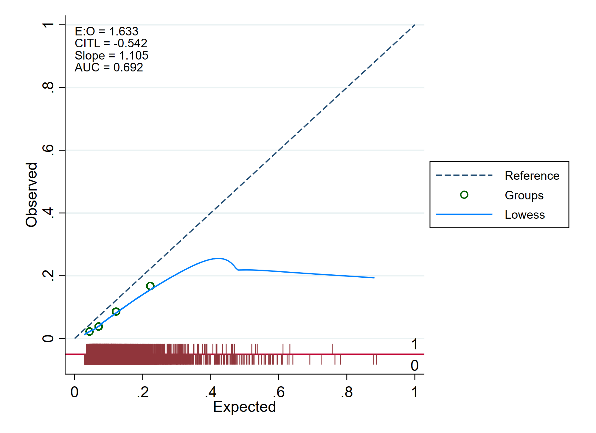


**SQLape®**


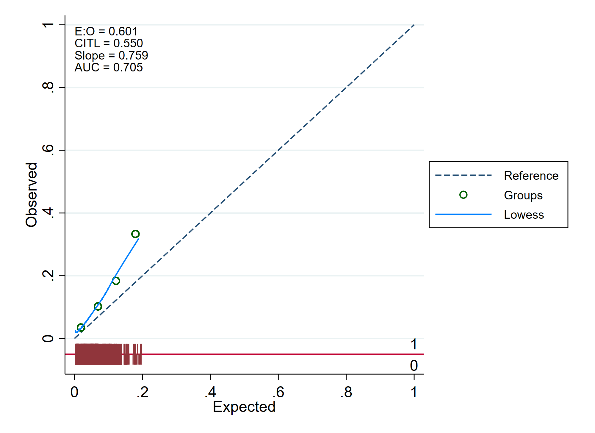


**LACE+**


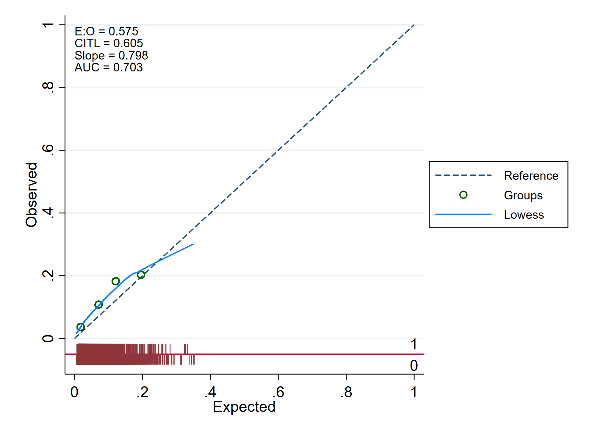

Supplement: S5 Appendix — (DOCX) [file pone.0258338.s005.docx]
