# Supplementary material for: External validation of EPIC’s Risk of Unplanned Readmission model, the LACE+ index and SQLape as predictors of unplanned hospital readmissions: A monocentric, retrospective, diagnostic cohort study in Switzerland
Source: PLoS One. 2021 Nov 12;16(11):e0258338. doi: 10.1371/journal.pone.0258338 (PMC8589185; doi:10.1371/journal.pone.0258338)
Supplement: S7 Appendix — (DOCX) [file pone.0258338.s007.docx]

# **S7. Appendix**

## **Reclassification tables – Cohort A**

SQLape® was considered as quasi standard:

**EPIC’s Risk of Unplanned Readmission**

**Cases** (N,%)

|  | **EPIC’s Risk of Unplanned Readmission** risk groups | | | | |
| --- | --- | --- | --- | --- | --- |
| **SQLape®** risk groups | No risk | Low risk | Medium risk | High risk | **Row Total** |
| No risk | 99 / 0.084 | 345 / 0.292 | 118 / 0.1 | 77 / 0.065 | 639 / 0.541 |
| Low risk | 9 / 0.008 | 133 / 0.113 | 144 / 0.122 | 150 / 0.127 | 436 / 0.369 |
| Medium risk | 3 / 0.003 | 17 / 0.014 | 28 / 0.024 | 46 / 0.039 | 94 / 0.080 |
| High risk | 0 / 0 | 1 / 0.001 | 5 / 0.004 | 6 / 0.005 | 12 / 0.010 |
| **Column Total** | 111 / 0.094 | 496 / 0.420 | 295 / 0.250 | 279 / 0.236 | **1181** |

**Controls** (N,%)

|  | **EPIC’s Risk of Unplanned Readmission** risk groups | | | | |
| --- | --- | --- | --- | --- | --- |
| **SQLape®** risk groups | No risk | Low risk | Medium risk | High risk | **Row Total** |
| No risk | 4771 / 0.218 | 10,628 / 0.485 | 1779 / 0.081 | 480 / 0.022 | 17,658 / 0.805 |
| Low risk | 96 / 0.004 | 1721 / 0.078 | 1264 / 0.058 | 754 / 0.034 | 3835 / 0.175 |
| Medium risk | 31 / 0.001 | 147 / 0.007 | 96 / 0.004 | 144 / 0.007 | 418 / 0.019 |
| High risk | 0 / 0 | 6 / 0.000 | 9 / 0.000 | 9 / 0.000 | 24 / 0.001 |
| **Column Total** | 4898 / 0.223 | 12,502 / 0.570 | 3148 / 0.144 | 1387 / 0.063 | **21,935** |

**LACE+**

**Cases** (N,%)

|  | **LACE+** risk groups | | | | |
| --- | --- | --- | --- | --- | --- |
| **SQLape®** risk groups | No risk | Low risk | Medium risk | High risk | **Row Total** |
| No risk | 507 / 0.429 | 86 / 0.073 | 33 / 0.028 | 13 / 0.011 | 639 / 0.541 |
| Low risk | 160 / 0.135 | 160 / 0.135 | 72 / 0.061 | 44 / 0.037 | 436 / 0.369 |
| Medium risk | 40 / 0.034 | 32 / 0.027 | 15 / 0.013 | 7 / 0.006 | 94 / 0.080 |
| High risk | 5 / 0.004 | 5 / 0.004 | 1 / 0.001 | 1 / 0.001 | 12 / 0.010 |
| **Column Total** | 712 / 0.603 | 283 / 0.240 | 121 / 0.102 | 65 / 0.055 | **1181** |

**Controls** (N,%)

|  | **LACE+** risk groups | | | | |
| --- | --- | --- | --- | --- | --- |
| **SQLape®** risk groups | No risk | Low risk | Medium risk | High risk | **Row Total** |
| No risk | 16,575 / 0.756 | 883 / 0.040 | 142 / 0.006 | 58 / 0.003 | 17,658 / 0.805 |
| Low risk | 1982 / 0.090 | 1330 / 0.061 | 356 / 0.016 | 167 / 0.008 | 3835 / 0.175 |
| Medium risk | 232 / 0.011 | 116 / 0.005 | 42 / 0.002 | 28 / 0.001 | 418 / 0.019 |
| High risk | 11 / 0.001 | 9 / 0.000 | 2 / 0.000 | 2 / 0.000 | 24 / 0.001 |
| **Column Total** | 18,800 / 0.857 | 2338 / 0.107 | 542 / 0.025 | 255 / 0.012 | **21,935** |
