# Supplementary material for: External validation of EPIC’s Risk of Unplanned Readmission model, the LACE+ index and SQLape as predictors of unplanned hospital readmissions: A monocentric, retrospective, diagnostic cohort study in Switzerland
Source: PLoS One. 2021 Nov 12;16(11):e0258338. doi: 10.1371/journal.pone.0258338 (PMC8589185; doi:10.1371/journal.pone.0258338)
Supplement: Attachment — Submitted filename: Response to Reviewer_PONE_D_21_01674R1.docx [file pone.0258338.s008.docx]

**Response to Reviewers**

This letter contains the author’s response to the PLOS ONE review from the 09.09.2021.

ID: PONE-D-21-01674R1

**Title:**

External validation of EPIC’s Risk of Unplanned Readmission model, the LACE+ index and SQLape® as predictors of unplanned hospital readmissions: A monocentric, retrospective, diagnostic cohort study in Switzerland

Submission due date for revised manuscript:

September 30th, 2021, 11:59 pm

## **GENERAL COMMENTS**

1) **Ethics Statement:** Please amend your current ethics statement in the Methods and online submission information to include the full name of the ethics committee/institutional review board(s) that approved your specific study. Only the waiver of informed consent is showing up as approved by the Ethics Committee Northwest- & Central Switzerland (EKNZ).

**Author`s response:** The statement was revised accordingly:

This study was approved by the Ethics Committee Northwest- & Central Switzerland (October 7, 2019, project-ID 2019-01861). An informed consent was not obtained, because this study was conducted as a quality control project that used anonymized data. This study was conducted according to the principles of the Declaration of Helsinki.
